# Body Composition and Nutrients Dietary Intake Changes during COVID-19 Lockdown in Spanish Healthy Postmenopausal Women

**DOI:** 10.3390/ejihpe12060047

**Published:** 2022-06-16

**Authors:** Cristina Acedo, Raul Roncero-Martín, Antonio Sánchez-Fernández, Cristina Mendoza-Holgado, María Pedrera-Canal, Fidel López-Espuela, Purificación Rey-Sánchez, Juan D. Pedrera-Zamorano, Luis M. Puerto-Parejo, Jose M. Moran, Jesús M. Lavado-García

**Affiliations:** 1 Metabolic Bone Diseases Research Group, Nursing Department, Nursing and Occupational Therapy College, University of Extremadura, 10003 Cáceres, Spain; cacedomo@alumnos.unex.es (C.A.); rronmar@unex.es (R.R.-M.); gineantonio@gmail.com (A.S.-F.); prey@unex.es (P.R.-S.); jpedrera@unex.es (J.D.P.-Z.); lmpuerto@unex.es (L.M.P.-P.); jmmorang@unex.es (J.M.M.); jmlavado@unex.es (J.M.L.-G.); 2 Health and Social Services Department, Government of Extremadura, 10001 Cáceres, Spain; cristina.mendoza@salud-juntaex.es; 3 Department of Nuclear Medicine, Hospital Clínico San Carlos, Av. Profesor Martín Lagos s/n., 28040 Madrid, Spain; mariapedreracanal@gmail.com

**Keywords:** body composition, COVID-19, lockdown, dietary intake

## Abstract

(1) Background: During the COVID-19 lockdown, high rates of physical inactivity and dietary imbalances were reported in both adults and adolescents. Physical separation and isolation not only have a significant impact on the performance of physical activity but also affect people’s lives, particularly their dietary habits. In the present study, we aimed to examine whether or not bioelectrical impedance-derived body composition parameters and dietary habits were affected during the pandemic-associated lockdown in postmenopausal Spanish women. (2) Methods: Sixty-six women participated in the study (58.7 ± 5.4 years) before (between July–October 2019) and after (August–October 2020) the lockdown, which occurred as a consequence of the COVID-19 pandemic in Spain. Body composition parameters were measured by bioelectrical impedance analysis while dietary intake of proteins, fat, carbohydrates, and energy was measured by a food frequency questionnaire. (3) Results Regarding body composition, no differences were observed in fat mass in % (mean increase 0.05 (2.74); *p* = 0.567), fat mass in kg (mean increase −0.07 (4.137); *p* = 0.356) or lean mass in kg (mean increase 0.20 (1.424); *p* = 0.636). Similarly, no statistically significant differences were observed between the two study periods for any of the nutrients studied, nor for energy intake (*p* > 0.05 in all cases). (4) Conclusions: After comprehensively assessing body composition and dietary intake of protein, fat, carbohydrates, and energy before and after COVID-19 lockdown in healthy adult women in Spain no changes in the parameters studied were observed during the period analyzed in the women examined.

## 1. Introduction

The aging process is characterized by sarcopenia (the age-related decline in skeletal muscle mass) and an accompanying rise in body fat mass. Sarcopenia has been closely linked to strength decline and also to functional decay, impairment, and decreased independence as age increases [1]. Menopausal transition accelerates general and abdominal obesity and sarcopenia, where lifestyle changes and insufficient levels of sexual hormones play a pivotal role [2]. This negative shift in the balance between fat and lean mass could be linked to a decrease in energy output and a decline in muscle strength [3,4].

According to current knowledge, keeping physically active throughout the years is essential to avoid negative age-related detrimental variations in body composition. Poor physical performance could be a sign of health problems related to aging [5]. Aerobic training with incremental aerobic workouts or resistance exercise represents an appealing choice for the elderly to improve and maintain their health [6,7]. The World Health Organization (WHO) officially classified coronavirus disease 2019 (COVID-19) as a pandemic on 11 March 2020, considering the rapid spread of the disease around the world [8].

Lacking effective treatments and vaccines, more than a hundred nations adopted restrictive policies in an effort to limit the spread and dissemination of the virus; consequently, by April 2020, over one-third of the global population was estimated to have been subjected to some form of mobility restriction [9]. During the COVID-19 lockdown [10], a high rate of physical inactivity and dietary imbalances were reported in both adults and adolescents [11]. Recent meta-analyses have shown that in a considerable majority of the participants there was an increase in body weight during lockdown [12]. Aging is associated with loss of muscle mass, decreased muscle strength, and decreased functional activity [13], which, due to confinement, might be exacerbated by a decrease in physical activity, aggravating it and even increasing mortality [14] and decreasing quality of life [15]. Maintaining regular physical exercise during a period of enforced rest, such as the ongoing coronavirus outbreak, is an essential preventive approach to physical and mental health [16].

Physical separation and isolation not only have a significant impact on the performance of physical activity but also affect people’s lives, particularly their dietary habits [17]. Hearing or reading about COVID-19 permanently in the media can be annoying and generate anxiety and stress. It causes people a strong desire to consume a specific food, called food “craving”. In Western societies, these foods are often highly palatable and energetic, which means that they include a lot of sugar and/or fat [18]. Even when people are bored, they eat more to distract themselves from the situation, especially if they have a high level of objective self-consciousness [19]. Consequently, maintaining a healthy and varied diet and regular physical activity may have been hindered by the lockdown.

Given that changes in body composition could have a negative impact on health, investigating the impact of the COVID-19 lockdown could provide a better knowledge of the body composition metrics mostly affected during a period of enforced inactivity in postmenopausal women. Therefore, the present study aimed to examine whether or not bioelectrical impedance-derived body composition parameters and dietary habits were affected during the pandemic-associated lockdown in postmenopausal Spanish women. We hypothesized that the lockdown had an impact on bioelectrical impedance-derived body composition parameters as well as on the intake of macronutrients.

## 2. Materials and Methods

### 2.1. Sample Characteristics

Sixty-six women participated in the study (58.7 ± 5.4 years) before (between July–October 2019) and after (August–October 2020) the lockdown, which occurred as a consequence of the COVID-19 pandemic in Spain. The women were part of a larger cohort study focused on the study of the analysis of the evolution of bone mass over 3 years, starting in 2019 with an expected completion date of 2022. The sample is of convenience (non-probabilistic) and corresponds to women for whom body composition data were available for the study period of interest. The study was performed in accordance with the Declaration of Helsinki and was approved by the Research Ethics Committee of the University of Extremadura. Written informed consent was obtained from all the subjects. Additionally, sociodemographic and clinical information was collected from the participants at the beginning and at the follow-up of the 12 months, including age, weight, body mass index (BMI), waist to hip ratio (WHR), and smoking habit. All the women were within the same ethnic group (Caucasian origin). All subjects led active lives.

### 2.2. Ethical Considerations

The Clinical Research Ethics Committee of the University of Extremadura approved this study (84/2018). All participants provided written informed consent; the study was performed in accordance with the Declaration of Helsinki.

### 2.3. Nutrients Intake

Total dietary carbohydrates, fats, proteins, and energy intakes were assessed via a 131-item food frequency questionnaire (FFQ). Food was quantified using a dietetic scale, measuring cups, cans, small bottles, and spoons, on the basis of current 7-day dietary records. This FFQ involved a 24-h recall performed over seven days [20]. The questionnaire used was self-reported, and the person completing the interview was blinded to the research question and hypothesis.

### 2.4. Anthropometric Study

Height was measured using a Harpenden stadiometer with a mandible plane parallel to the floor, and weight was measured using a biomedical precision balance. Height was measured to the nearest cm and weight to the nearest 100 g. Both measurements were determined when the participants were wearing only light clothing and no shoes. BMI was calculated as the weight in kilograms divided by the square of the height in meters (kg/m^2^). The WHR was calculated by dividing the waist circumference by the hip circumference. Waist and hip circumference were measured according to World Health Organization (WHO) recommendations. We asked subjects to remain relatively relaxed with their arms at their sides, feet placed together, and weight evenly balanced between the feet. The waist circumference was measured midway between the lowest rib and the upper edge of the iliac crest. Hip circumference was measured at the widest part of the buttocks (trochanters). Obesity based on both BMI (≥30 kg/m^2^) and WHR (≥0.85 for women) was defined in accordance with WHO guidelines. Patients were divided into specific WHR tertiles some of the calculations performed in the study.

### 2.5. Body Composition Measurements Using Bioelectrical Impedance Analysis (BIA)

BIA was performed in the early morning after an overnight fast of at least 12 h. BIA was measured in subjects wearing light-colored clothes and standing erect with their bare feet on the analyzer footpads. Feet were cleaned with soap and water and air-dried prior to the BIA procedure. A Tanita BC-418 MA Segmental Body Composition Analyzer (Tanita Corp., Tokyo, Japan) was used. Specific data for body composition calculations included age, sex, and body type (athletic, average). The analyzer measurements were performed with eight polar electrodes: two rectangular stainless steel electrodes at the base of the system, attached to a metal platform placed over force transducers for weight measurement, and limb grip electrodes with anterior and posterior portions. The eight electrodes were connected to a digital circuit board that electronically switched the electrical circuit under study. This device provided further data on weight, total fat percentage (FP), fat mass (FM), fat-free mass (FFM), and total body water (TBW). The use of this system in the determination of body composition was validated against the DXA method in healthy adults [21].

### 2.6. Statistical Analysis

IBM-SPSS Version 24 statistical software (SPSS version 24.0, SPSS Inc. Chicago, IL, USA) was used for analysis. The data were characterized descriptively by the mean and standard deviation or by median and interquartile range. Kolmogorov-Smirnov tests were used to assess the normality of the data distribution. Based on the distribution of the data, nonparametric methods were used (Wilcoxon test). A two-sided *p* value < 0.05 was considered statistically significant.

## 3. Results

**Figure 1:**
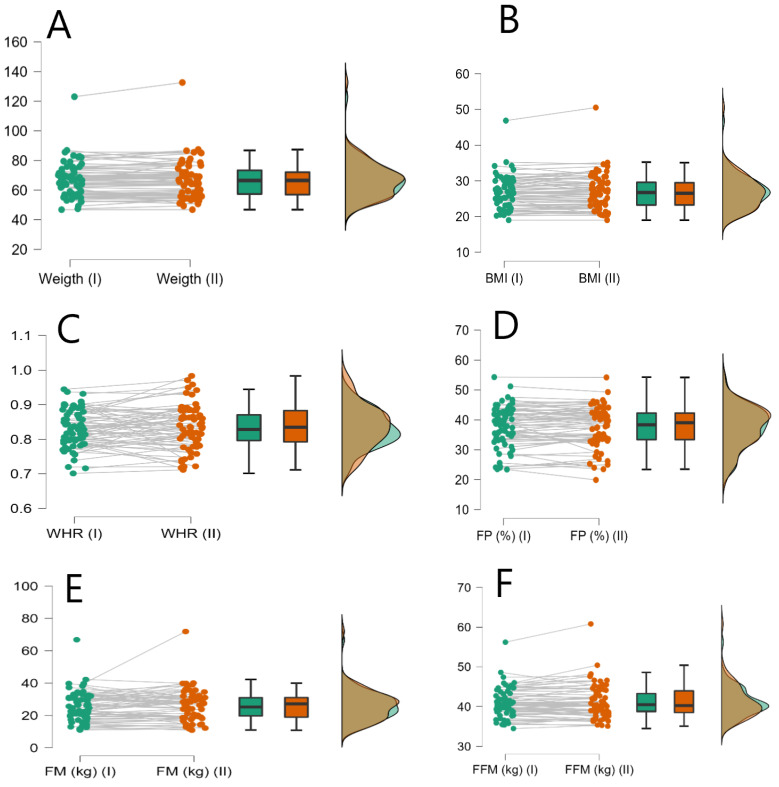
Raincloud plot for the individual data of the study participants. Panel (**A**), weight, Panel (**B**) BMI (Body Mass Index), Panel (**C**) (WHR) Waist Hip Ratio, Panel (**D**) (FP) Fat Mass Percentage, Panel (**E**) (FM) Fat Mass in Kilograms, Panel (**F**) (FFM) Fat Free Mass in Kilograms.

The mean age of the participants at the beginning of the study was 57.8 years (SD = 4.3). A mean weight increase of 0.44 (3.68) kg was recorded, which was not statistically significant (*p* = 0.243). An increase in BMI in the studied sample was also observed in the period of study 0.15 (1.427), which was also not statistically significant (*p* = 0.338). Other anthropometric measures such as WHR (mean increase 0.0052 (0.05)) did not show statistically significant differences either (*p* = 0.269). Regarding body composition, no differences were observed in fat mass in % (mean increase 0.05 (2.74); *p* = 0.567), fat mass in kg (mean increase −0.07 (4.137); *p* = 0.356) or lean mass in kg (mean increase 0.20 (1.424); *p* = 0.636) (Table 1). The change in key parameters analyzed was calculated for the period studied to evaluate possible trends in their evolution. All variables showed a positive increment except for lean mass. The individual trend for each of the variables of interest during the study period for each of the participants in the study is illustrated in Figure 1.

Based on the BMI of the participants, a total of *n* = 14 (21.1%) of the women were obese, *n* = 28 overweight (42.4%), and 24 normal (36.4%) at the beginning of the study, and 15 (22.7%) were obese, 26 overweight (39.4%), and 25 normal (37.9%) after the lockdown period due to COVID-19. After stratifying the participants according to their BMI prior to confinement, no differences were observed in the evolution of weight (*p* = 0.692), BMI (*p* = 0.749), fat mass in % (*p* = 1), fat mass in kg (*p* = 0.875) and lean mass (0.715) in the group of women with normal BMI. In the group of overweight women, no differences were observed in any of the aforementioned parameters (*p* > 0.05 in all cases). Finally, no statistically significant differences in the previously studied parameters were observed in the group of obese women (*p* > 0.05 in all cases) (Table 2).

We continued to investigate possible changes in major nutrient intake that may have occurred during the confinement period. Protein (g/day), fat (g/day), carbohydrate (g/day), and energy (kcal/day) intake were analyzed (Table 3).

No statistically significant differences were observed between the two study periods for any of the nutrients studied, nor for energy intake (*p* > 0.05 in all cases). After extending the analysis based on BMI, no significant differences were observed in the dietary parameters studied (Table 4).

## 4. Discussion

In the present study, we have analyzed the evolution of body composition and nutrient intake in healthy postmenopausal women during the quarantine period due to the COVID-19 outbreak in Spain. Our hypothesis that lockdown had an impact on bioelectrical impedance-derived body composition parameters, as well as macronutrient intake in the women studied, could not be verified. Spain was one of the most affected countries in Europe during the first outbreak of COVID-19. Due to the measures adopted to prevent the spread of the virus, the Spanish government adopted strict containment measures that forced Spanish citizens to experience a period of quarantine, which prevented most citizens from leading normal lives from March to June 2020.

Similar results have been reported in professional athletes who had to suspend their regular training season due to confinement [22]. However, in another study conducted with soccer players, the study of body composition did report an increase in FM% during the lockdown period; however, in this study, fat mass was studied by skinfolds and not by electrical bioimpedance, which could explain the difference in the results [23]. In Spanish children, a considerable impact on parameters such as BMI has also been reported, but no differences were reported in waist measurements during the period [24]. It has also been reported that overweight and obese subjects gained body mass, while underweight subjects lost body mass during the lockdown [25], which was not observed in our study.

Our results are consistent with those published in healthy adults and are associated with no change in body composition (even in a context of low physical activity due to lockdown) [26]. Conversely, the results reported here are contrary to those obtained in a sample of similar size (*n* = 51), but of younger age, in which statistically significant differences in BMI were observed, reporting a moderate increase in the parameter but without exceeding the recommended healthy values [27].

In the rural population of Italy, and specifically in women belonging demographically to a group more similar to our sample, statistically significant differences were observed in the dietary intakes of the main groups of macronutrients during the quarantine [28], data that come from a significantly larger sample than ours and that could explain the greater precision in detecting changes in dietary patterns during the study period. In adults from Slovenia, significant differences in protein intake (decrease) and increase in saturated fat intake have been reported for a research period similar to the one we have reported [26].

In the present study, we recognize different limitations that may affect the conclusions derived from it. First, the non-probabilistic nature of the sample analyzed does not allow us to generalize the data obtained to the population from which it was taken. Secondly, the small sample size analyzed, and the negative result obtained, could be related to a type II error phenomenon, and therefore the lockdown period may have had an effect on the factors analyzed but this could not be detected due to a lack of statistical power. Finally, in the present study, it was not possible to control for other potential confounding factors such as teleworking that could allow individuals, couples, and families to have and share healthy home-cooked meals more frequently and other variables that could affect diet such as relationship status, family members, occupation, etc.

## 5. Conclusions

The novelty of this study is that, to the best of our knowledge, it is the first to comprehensively assess body composition and dietary intake of protein, fat, carbohydrates, and energy before and after the COVID-19 lockdown in healthy adult women in Spain. No changes in the parameters studied were observed during the period analyzed in the women examined.

## Figures and Tables

**Table 1:** Anthropometric and body composition parameters measured.

	Before COVID-19 Lockdown	After COVID-19 Lockdown		Pre/Post Change
	(*n* = 66)	(*n* = 66)		
	Median (IQR)	Median (IQR)	*p*	Median IQR
Weight (kg)	66.45 (15.97)	66.55 (15.12)	0.243	0.6 (3.45)
BMI	26.72 (6.38)	26.52 (6.24)	0.338	0.20 (1.38)
Waist (cm)	85 (14)	87 (14.75)	0.356	1(6.75)
Hip (cm)	104 (12.75)	104 (13.5)	0.458	1 (5)
WHR	0.8283 (0.07)	0.8348 (0.09)	0.269	0.006 (0.06)
FP (%)	38.35 (8.9)	39.05 (8.9)	0.567	0.45 (3.2)
FM (kg)	25.15 (11.15)	27.1 (12)	0.356	0.75 (3.25)
FFM (kg)	40.45 (4.5)	40.25 (5.4)	0.636	−0.1 (1.5)

Comparisons between the periods studied were made using the Wilcoxon test.

**Table 2:** Anthropometric and body composition measurements according to WHO BMI classification.

	Normal (*n* = 24)			Overweight (*n* = 28)			Obese (*n* = 14)		
	Before COVID-19 Lockdown	After COVID-19 Lockdown		Before COVID-19 Lockdown	After COVID-19 Lockdown		Before COVID-19 Lockdown	After COVID-19 Lockdown	
	Median (IQR)	Median (IQR)	*p*	Median (IQR)	Median (IQR)	*p*	Median (IQR)	Median (IQR)	*p*
Weight (kg)	56.1 (6.25)	55.3 (5.225)	0.692	67.55 (6.87)	68.15 (6.87)	0.172	77.7 (8.82)	77.55 (13.95)	0.583
BMI	22.17 (1.98)	22.27 (2.47)	0.749	27 (1.69)	27.54 (2.31)	0.255	31.24 (3.14)	32.02 (2.58)	0.583
Waist (cm)	77.5 (8.75)	78 (11.5)	0.794	88 (8.5)	88 (8.25)	0.243	99 (11)	99.5 (7.5)	0.801
Hip (cm)	94.5 (8.5)	96 (5.5)	0.355	105 (6)	105 (7.5)	0.213	116 (7.25)	115.5 (10.25)	0.182
WHR	0.801 (0.057)	0.795 (0.08)	0.867	0.832 (0.06)	0.841 (0.05)	0.745	0.859 (0.05)	0.873 (0.07)	0.173
FP (%)	31.4 (7.32)	32.9 (7.3)	1	39.25 (5.57)	40.55 (4.87)	0.16	44.55 (3.82)	44.25 (3.85)	0.286
FM (kg)	17.3 (6.22)	18.4 (5.12)	0.715	26.5 (7.4)	27.7 (6.15)	0.741	33.25 (6.82)	33.3 (7.77)	0.102
FFM (kg)	38.55 (4.25)	38.4 (3.6)	0.875	40.75 (3.85)	40.65 (4.97)	0.141	43.8 (5.32)	44.7 (7)	0.855

Comparisons between the periods studied were made using the Wilcoxon test. Normal BMI = 18.5–24.9, Overweight BMI = 25.0–24.9; Obese BMI ≥ 30.

**Table 3:** Dietary and energy intake.

	Before COVID-19 Lockdown	After COVID-19 Lockdown	
	*n* = 63	*n* = 58	
	Median (IQR)	Median (IQR)	*p*
Proteins (g/day)	88.38 (36.96)	89.96 (39.95)	0.943
Fat (g/day)	81.2 (45.61)	79.15 (40.78)	0.589
Carbohydrate (g/day)	278.4 (138)	254.9 (108)	0.688
Energy (kcal/day)	2235 (788.1)	2184 (888.3)	0.818

Comparisons between the periods studied were made using the Wilcoxon test.

**Table 4:** Anthropometric and body composition measurements according to WHO BMI classification.

	Normal			Overweight			Obese		
	Before COVID-19 Lockdown (*n* = 23)	After COVID-19 Lockdown (*n* = 23)		Before COVID-19 Lockdown (*n* = 28)	After COVID-19 Lockdown (*n* = 24)		Before COVID-19 Lockdown (*n* = 12)	After COVID-19 Lockdown (*n* = 11)	
	Median (IQR)	Median (IQR)	*p*	Median (IQR)	Median (IQR)	*p*	Median (IQR)	Median (IQR)	*p*
Proteins (g/day)	94.77 (35.41)	96.69 (56.14)	0.424	84.59 (35.83)	90.19 (31.63)	0.989	85.09 (47.10)	77.05 (25.39)	0.465
Fat (g/day)	72.95 (48.85)	82.87 (41.3)	0.656	81.54 (29.37)	81.08 (24.98)	0.700	81.23 (52.61)	66.67 (48.95)	0.32
Carbohydrate (g/day)	287.5 (142.9)	242.5 (144.6)	0.679	289.3 (129.4)	289.4 (100.3)	0.668	240.9 (122)	257.5 (44.45)	0.700
Energy (kcal/day)	2363 (1044)	2199 (1078)	0.975	2249 (593.3)	2269 (689.6)	0.812	2040 (644.4)	1988 (394.5)	0.365

Comparisons between the periods studied were made using the Wilcoxon test. Normal BMI= 18.5–24.9, Overweight BMI = 25.0–24.9; Obese BMI ≥ 30; IQR (Interquartile range).

## Data Availability

The dataset analyzed during the current study is not publicly available due to national data regulations and for ethical reasons, including that we do not have the explicit written consent of the study volunteers to make their deidentified data available at the end of the study. However, datasets and SPSS statistical analyses can be requested by sending a letter to the corresponding author.
